# Low-Level Laser Therapy in Prevention of the Development of Endothelial Dysfunction and Clinical Experience of Treatment and Rehabilitation of COVID-19 Patients

**DOI:** 10.1155/2021/6626932

**Published:** 2021-01-26

**Authors:** Sergey Moskvin, Evgeniy Askhadulin, Andrey Kochetkov

**Affiliations:** ^1^O.K. Skobelkin State Scientific Center of Laser Medicine Under the Federal Medical Biological Agency, Studencheskaya str., 40, Moscow 121165, Russia; ^2^Center for the Treatment of Patients with COVID-19, “Outpatient Clinic of Rassvet Settlement”, Rassvet settlement, 38, Tula 301212, Russia; ^3^Central Clinical Hospital for Rehabilitation Under the Federal Medical Biological Agency, Russia

## Abstract

**Objectives:**

The aim of the article is to justify the application of low-level laser therapy (LLLT) to prevent the development of endothelial dysfunction in COVID-19 patients. The results of treating and rehabilitating patients with COVID-19 and prevention of the disease using low-level laser therapy (LLLT) are evaluated.

**Methods:**

A literature review is conducted on mechanisms of vascular homeostasis regulation, biomodulating effect of laser light, and LLLT methods for preventing endothelial dysfunction. A total of 106 patients were treated in two COVID-19 healthcare centers in Russia. 22 patients with SARS (+) pneumonia at the stage of resolving the pathological lesion were admitted to rehabilitation using pulsed IR laser. 14 patients with acute forms of COVID-19 were treated using LASMIK device: wavelength 904 nm, pulsed mode, externally and ILBI-525 (intravenous laser blood illumination) + LUVBI (ultraviolet laser blood illumination). 70 persons underwent preventive courses of noninvasive LLLT.

**Results:**

It was shown that LLLT is effective in preventing the development of endothelial dysfunction. Clinical experience demonstrated good tolerability of the treatment, improvement in sputum discharge, and an improvement in overall health. The severity of general hypoxia decreased by the 5th procedure. The procedures for prevention of the disease were well tolerated; there were no cases of COVID-19.

**Conclusion:**

Low-level laser therapy is a justified treatment method that promotes lung tissue regeneration and mitigates the consequences of the disease. The obtained results confirm that LLLT can be used for the effective prevention and treatment of COVID-19 patients.

## 1. Introduction

The coronavirus disease 2019 (COVID-19), a global pandemic caused by SARS-CoV-2, has become a challenge for all mankind, primarily for scholars and doctors, who are tasked with finding possible ways for disease control, effective treatment of patients with minimization of mortality and development of complications, and the rehabilitation of patients. In humans, this viral infection can cause a number of diseases, including severe acute respiratory syndrome [1].

The apparent nonspecificity of the observed lesions in various organs and systems of physiological regulation is one of the numerous features of COVID-19. Simultaneously, the development of endothelial dysfunction (EnD) can be distinguished as a factor that is largely associated with various disorders. Many experts are convinced that the vascular endothelium is the cornerstone of organ dysfunction in severe SARS-CoV-2 infection [2].

In patients who died from COVID-19-associated respiratory failure, diffuse alveolar injury with perivascular T-cell infiltration was the histological pattern in the peripheral lung. The lungs of these patients showed distinctive vascular peculiarities, including severe endothelial injury associated with the presence of intracellular virus and destroyed cell membranes. Histological analysis of pulmonary vessels in patients with COVID-19 showed widespread thrombosis with microangiopathy. Alveolar capillary microthrombi were nine times more common in patients with COVID-19 than in those with influenza (*p* < 0.001). All these factors indicated the development of severe EnD [3].

The endothelial dysfunction (EnD) is a complex multifaceted process and a rather serious problem in modern clinical practice, even if not considered in the context of COVID-19 [4]. However, in case of a viral infection, studying the possibility of preventing the development of this pathology is of particular paramount importance. The endothelium performs multiple functions such as regulation of transporting many biologically active substances; barrier; participation in phagocytosis; secretory function; and control of fluid diffusion, electrolytes, metabolic products, platelet adhesion, and aggregation. Therefore, the EnD can be catastrophic, becoming the primary cause of high mortality and the development of serious complications that disrupt a full-fledged human life.

Concomitant diseases can divide and synergistically activate pathophysiological pathways. Thus, inflammation activates cerebrovascular pathology through proinflammatory cytokines, endothelin-I, and nitric oxide (NO), which contributes to a long-term change in the structure of fatty acids, proteins, DNA, and mitochondria. Occurring dysfunctional energy metabolism (impaired production of mitochondrial ATP), formation of amyloid-*β*, development of the EnD, and impaired permeability of the blood-brain barrier result in decreased cerebral blood flow and chronic cerebral hypoperfusion, which modulates metabolic dysfunction and neurodegeneration. In fact, the brain is deprived of oxygen and nutrients and suffers from synaptic dysfunction and degeneration/loss of neurons, leading to gray and white matter atrophy, cognitive dysfunction, and the development of Alzheimer's disease. Consequently, eliminating inflammation is the main goal of the therapeutic effect for the restoration of reduced cerebral blood flow and hypometabolism [5].

The violation of microcirculation and associated metabolism of lung parenchyma is one of the main pathomorphological mechanisms of COVID-19 pneumonia development. The main goals of rehabilitation for patients with pneumonias include restoring respiratory function (RF), relieving and preventing the development of a premature airway closure syndrome, eliminating dissociation between alveolar ventilation and pulmonary perfusion, and restoring bronchial conduction and full thoracic excursions [6].

Unfortunately, so far, no sufficiently effective methods for disease prevention, treatment, and rehabilitation of COVID-19 patients have been put forward, and mortality remains quite high. Many different options have been proposed, among which the prospects for low-level laser therapy (LLLT) are being discussed rather actively [7–9].

It is known that the thermodynamic triggering of intracellular Ca^2+^-dependent processes as a result of laser light energy absorption underlies the primary mechanism of the biomodulating effect of low-intensity laser illumination (LILI), followed by further development of secondary responses of the body, restoring (normalizing) the work of almost all systems that regulate and maintain homeostasis: the immune, nervous, circulatory, and hormonal systems [10–12].

The following LILI properties are most interesting in terms of treating and rehabilitating patients with pneumonia: enhanced enzymatic activity in the mitochondrial respiratory chain, lipid peroxidation system, increased RBC hemoglobin-oxygen affinity, increased functional activity of tissue macrophages, and improved functioning of the actin-myosin complex. At the systemic level, these features are manifested by the activation of microcirculation and metabolism, improved regeneration of lung tissue, enhanced muscle support for the respiratory act, and increased local immunity [13].

### 1.1. Molecular-Cellular and Physiological Mechanisms for Vascular Homeostasis Regulation

A violation of the bioavailability of nitric oxide (NO) through the suppression of endothelial NO synthetase (NOS) and the resulted decrease in the synthesis of NO are the main manifestations of EnD [14]. Under physiological conditions, there is an equilibrium between the vasoconstrictors secreted by the endothelium and the vasodilators. The violation of this equilibrium leads to a local spasm and an increase in vascular tone. As a result, gradual depletion and perversion of the compensatory capacity of the endothelium may occur, leading to a violation of the rather complex regulation of the natural mechanisms for expansion and narrowing of the vascular bed [15].

The endothelium plays a key role in maintaining vascular homeostasis through the release of biologically active substances (Table 1), but is also susceptible to the external regulators [16–18]:

- mast cells that release heparin and histamine
- platelets containing vascular endothelium growth factors and blood coagulation factors, etc.
- hormones and neuropeptides (adrenaline, acetylcholine, histamine, bradykinin, neurourethric peptides, etc.)

As summarized by Suchkov (2012) [4], the possible ways for pharmacological correction of EnD, despite the known regulatory mechanisms (Table 1), require further comprehensive study and evaluation in view of low efficiency and adverse side effects. Physiotherapeutic procedures are considered as one of the options for normalizing the functional state of the endothelium [19].

### 1.2. Primary and Secondary Mechanisms for the Biomodulating Effect of Low-Intensity Laser Illumination (LILI)

**Figure 1:**
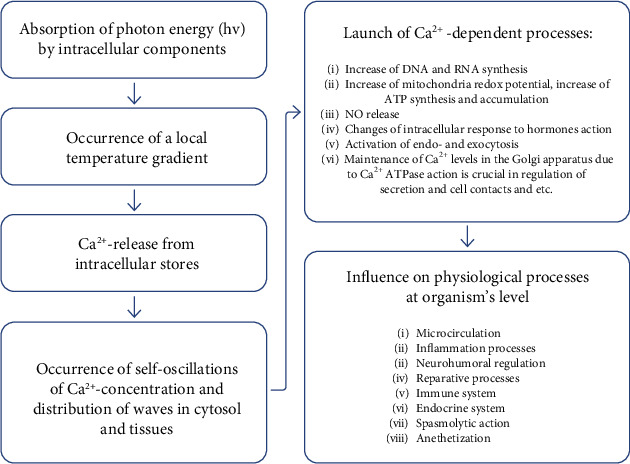
Biological effects of LLLT.

According to the current opinions, which are in good agreement with the practice of clinical application of low-level laser therapy, thermodynamic triggering of Ca^2+^-dependent processes is the primary mechanism of the biomodulating effect of LILI. After the photon energy (laser light) absorption by various intracellular components, the intracellular calcium depot is activated, and Ca^2+^ ions with increased concentration are released in the form of two waves with half periods of 100 and 300 seconds. This is followed by the development of a cascade of responses at all levels, from cells to the body as a whole: activation of mitochondrial functions, cell metabolism and proliferation, normalization of the immune and vascular systems, and the inclusion of the VNS and CNS in the process, etc. (Figure 1) [20–22].

The versatility and high efficiency of low-level laser therapy (a unique physiotherapeutic method in many respects) are explained precisely by the exposure to the maximum frequency of electromagnetic waves (optical range) at the cellular level and the coherence (monochromaticity) of laser light.

### 1.3. Impact of LILI on the Factors Maintaining Vascular Homeostasis

It is well known that the activity of practically all the aforementioned regulators (Table 1) is associated with a change in the concentration of Ca^2+^ ions to a certain extent. Therefore, we will refer to only a few reviews instead of citing numerous publications [23, 24].

From the viewpoint of the research topic, we are primarily interested in nitric oxide and its synthesis and release, which is a Ca^2+^-dependent process [25]. So it is not surprising that many studies confirm the ability of LILI to stimulate NO release, thereby ensuring vascular homeostasis regulation [26–32]. Moreover, in some studies, the authors have demonstrated a direct relationship between the increased intracellular concentration of Ca^2+^ and the intensity of NO release as well as the subsequent vasodilation [33–35].

The normalization of the endothelial system in children suffering from bronchial asthma was confirmed by changes in various parameters of blood plasma, including Endothelin-1 and nitric oxide content [36, 37].

The ability of LILI to effectively stimulate the release of PGE2 has been known for a long time, and it has been shown experimentally [38–40] and in the clinic [41–43].

It is known that the course use of both external low-level laser therapy with pulsed infrared LILI and intravenous laser blood illumination (ILBI) in patients with arterial hypertension improves a number of biochemical, hemorheological, and hormonal parameters (C-peptide, insulin, angiotensin, bradykinin, aldosterone, and cortisol), and the results are preserved for up to six months [44–46].

Many authors have demonstrated the role of the kallikrein system in hemovascular regulation and the possibility of correcting it through the blood illumination with a laser red light (wavelength 635 nm) and/or incoherent ultraviolet (UV) light [47–50].

The anti-inflammatory effect of LILI has been well-researched and in great detail; this property of laser light is perhaps most actively used in current low-level laser therapy [22]. Thus, it is not necessary to provide examples of, literally, thousands of studies on this topic, and anyone can obtain additional comprehensive information by requesting it from the authors of the articles.

### 1.4. Low-Level Laser Therapy Methods for Vascular Pathologies of Varying Origins

If we refer specifically to COVID-19, it is mandatory to employ noninvasive (percutaneous) or intravenous laser blood illumination (NLBI or ILBI); additionally, the immunocompetent organs and lesion projections are exposed to laser light [51]. This approach combining systemic and local exposure to LILI has shown itself to be most efficacious in clinical practice [52–54].

Intravenous laser blood illumination has long been a well-established method for correcting endothelial function. Most often, the “classic” version is used: 635 nm wavelength, 2–3 mW illumination power at the fiber output, 10–20 min exposure [55–61], but recently, a combined version of the technique involving laser ultraviolet blood illumination (LUVBI®) has been used increasingly [62–64].

Furthermore, experts are well aware of how effective it is to combine low-level laser therapy with other physiotherapeutic methods [65]; however, this topic is beyond the scope of the present article.

### 1.5. Clinical Experience

In Russia, low-level laser therapy is a standard procedure that is included in temporary methodological guidelines “Prevention, Diagnosis and Treatment of New Coronavirus Infection (COVID-19),” meaning that this method is officially permitted. There are dozens of COVID-19 centers using low-level laser therapy.

We present the results of the practical work of two Russian healthcare centers for COVID-19 patients. The subjects were not specially selected, all patients were treated with standard protocols, and the effect was assessed by clinical results.

During the period from April 01, 2020, to June 15, 2020, in one of these centers (in St. Petersburg), 22 patients who were diagnosed with SARS (+) pneumonia of varying severity and mainly without respiratory failure or with type I respiratory failure at the stage of resolving the pathological lesion according to computed tomography (CT) were treated. The condition for admitting such patients to rehabilitation (in accordance with the recommendations of the Ministry of Health of the Russian Federation) consisted of two available negative smears.

At the initial examination, 90% of the patients presented nonspecific complaints characterizing the presence of hypoxia (asthenization, malaise, and sweating); approximately one-third of the patients had complaints of inspiratory dyspnea, and about 5% complained of a feeling of incomplete inhalation or difficulty in exhaling. According to the physical examinations, chest breathing—which is less physiologically beneficial than other types of breathing—was revealed in the majority of patients; the amplitude of thoracic excursions was decreased, and an auscultatory picture corresponded to the stage of pneumonia resolution.

In addition to breathing exercises, simulator exercises, vibration gymnastics, and aerosol therapy, the rehabilitation scheme for all patients was supplemented with a course of low-level laser therapy (“Matrix” device). The exposure was carried out with a pulsed IR LILI (904 nm wavelength, 100 ns light pulse duration, 15 W pulsed power, 80 Hz frequency, and 1.5 min exposure time per one zone) on the following skin projection areas:

- focus of inflammation (2–3 zones)
- pulmonary hilum
- Kronig isthmus
- left supraclavicular region (noninvasive laser blood illumination, NLBI)

It was recommended to carry out 12–15 procedures per course daily or every other day.

All patients demonstrated good tolerability of the treatment; after the second procedure, there was an improvement in sputum discharge due to an increase in the effectiveness of the cough push and an improvement in overall health. The severity of general hypoxia decreased by the 5th procedure. By the end of the rehabilitation course, a complete regression of complaints was recorded in 90% of the patients. After the completion of the course, it was recommended to continue the autonomous breathing exercises to increase the functional pulmonary reserves and maintain their performance at the highest possible level.

Comparison of the results was carried out in patients with COVID-19 who are in rehabilitation after the main treatment. There were 10 subjects (5 men and 5 women) in each group; randomization was carried out by the method of sequential numbers. The average age of patients in the 1st group was 52.8 ± 10.4 years, and in the 2nd group—53.1 ± 10.7 years.

Patients of the 1st group administered standard procedures (exercise therapy, etc.) according to the recommendations [6, 66], and in the 2nd group, standard procedures were combined with low-level laser therapy.

The results were evaluated before the beginning, after 5 and 15 days of the course of treatment. The assessment of the patient's condition, according to the clinical guidelines [66], was carried out according to the Borg scale [67] and MRC (dyspnea) [68].

To analyze the obtained data, we used Microsoft Office Excel (2013) with a package of statistical data analysis. Statistically significant differences in indicators were considered confirmed at a significance level of *p* < 0.05.

**Figure 2:**
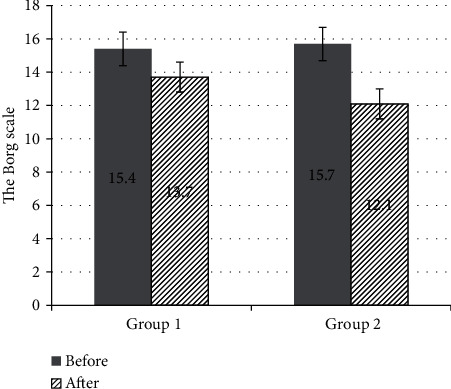
The mean scores before and after the treatment in groups 1 and 2 according to the Borg scale.

**Figure 3:**
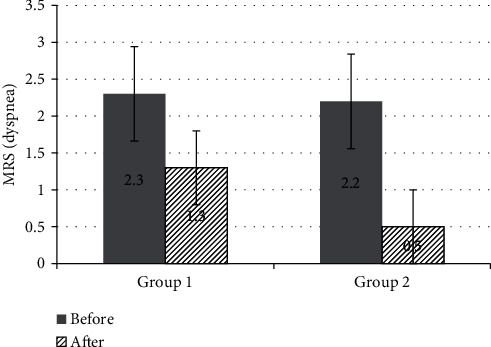
The mean scores before and after the treatment in groups 1 and 2 according to the MRS scale (dyspnea).

The results are shown in Figures 2 and 3 (comparative graphs).

The groups did not significantly differ in terms of the severity of the disease; the mean score on the Borg scale in group 1 before the beginning of treatment was 15.4 ± 1, and in group 2—15.7 ± 0.9, the mean score on the MRS scale (dyspnea) in group 1 was 2.3 ± 0.5, and in group 2—2.2 ± 0.6.

After treatment, the mean score on the Borg scale in group 1 decreased to 13.7 ± 1.1 and in group 2 to 12.1 ± 0.7, the mean score on the MRS scale (dyspnea) was 1.3 ± 0.6 and 0.5 ± 0.5, respectively. An important result is that according to the Borg scale, the indicator significantly decreased by ≥2 points in 7 people in group 1, and in group 2—in 10 people; according to the MRS scale, the indicator significantly decreased by ≥1 point in 6 people in group 1, and in group 2—10 people. That is, in all patients who underwent rehabilitation with low-level laser therapy, exercise tolerance significantly increased, and shortness of breath significantly decreased or disappeared.

Reduction in the rehabilitation period was assessed by the time of the onset of the first clinical improvements in 50% of patients with a 1-point change in the Borg score. This indicator averaged 5.6 days in the 1st group, and 3.2 days in the 2nd group.

In the period from April 01, 2020, to June 30, 2020, 14 patients with a confirmed positive smear were treated in the Tula Health Care Center, and in two patients, this diagnosis was confirmed with a CT scan. At the initial examination, 90% of the patients complained of shortness of breath with little physical exertion, cough, malaise, general weakness, sweating, and anosmia.

The patients with mild disease (six persons, including children from the family of a sick employee) underwent seven noninvasive LLLT procedures daily (the technique is described above). The treatment was well tolerated by everyone; after the first procedure, there was a relief of chest pain during coughing, improvement in sputum discharge due to an increase in the effectiveness of the cough push, and an improvement in overall health. The phenomena of intoxication and general hypoxia decreased by the fifth procedure, and the temperature returned to normal.

In one case, the course of the disease was assessed as severe, requiring long-term hospitalization with a diagnosis of SARS (+) bilateral pneumonia with type II–III respiratory failure. The course of postdischarge rehabilitation consisted of five daily procedures of combined laser therapy (LASMIK device): intravenous laser blood illumination ILBI-525 + ultraviolet laser blood illumination LUVBI® (525 nm wavelength, green spectrum, 2 mW illumination power, 5 min exposure per one zone + 365-nm wavelength, UV spectrum, 2 mW illumination power, and 5 min exposure per zone on alternate days) and exposure to pulsed IR LILI (904 nm wavelength, 100 ns light pulse duration, 15 W pulsed power, 10–15 W/cm^2^ power density, 80 Hz frequency, and 1.5 min exposure time per exposed zone) on the following skin projection areas: the inflammation foci in the lung tissue (2–3 zones), the pulmonary hilum, and the left supraclavicular region. After the first procedure, the patient already noted a decrease in fatigue, general weakness, reduced (“now and then”) coughing, relief of sputum discharge, and an improvement in overall health. By the fifth procedure, she noted a significant improvement in overall health and the disappearance of shortness of breath with moderate physical exertion.

During the same period, with regard to an outbreak of the disease among health workers (with a fatal case before the start of low-level laser therapy) and to prevent the development of fatal complications, the employees of two medical institutions (70 persons) underwent preventive courses of noninvasive low-level laser therapy (3–5 procedures daily or every other day). They were exposed to a pulsed IR LILI (see above the parameters of the technique) on the area of the skin projection of the pulmonary hilum and left supraclavicular region. All procedures were well tolerated by everybody; no cases of COVID-19 were detected. Moreover, other simultaneous positive results were observed. After the first procedure, health workers who have had concomitant diseases for several years (bronchial asthma, chronic obstructive bronchitis, allergic rhinitis, etc.) noted an improvement in overall health and relief of the symptoms of chronic diseases. Furthermore, after the fifth procedure, the improvement was so significant that we could speak about real treatment. Although the effectiveness of laser therapy in the bronchopulmonary pathology of various origins is quite well demonstrated [12, 69], unfortunately, this method remains in insufficient demand.

## 2. Conclusion

In our opinion, the presented brief literature review convincingly demonstrates the possibilities of low-level laser therapy for eliminating EnD. LLLT is a pathogenetically justified treatment method that promotes lung tissue regeneration and mitigates the consequences of the disease.

Moreover, there is already a positive experience of applying low-level laser therapy for the comprehensive treatment and rehabilitation of COVID-19 patients [51, 70].

We will continue to evaluate the effect of low-level laser therapy for prophylactic and therapeutic purposes among medical staff during the pandemic in the long term—after the next 3, 6, and 9 months. We believe that the experience gained confirms the correctness and equity of including low-level laser therapy in the Russian clinical guidelines. There is also full confidence that the method can be used for the effective prevention and treatment of COVID-19 patients.

For more detailed information, and the results of further work, please contact the authors by the e-mail 7652612@mail.ru.

## Figures and Tables

**Table 1:** Physiologically active substances and the circulatory system regulators synthesized in the endothelium.

*Vascular tone regulators*	
Vasoconstrictors	Vasodilators
Endothelin-1 and -2 Angiotensin II Thromboxane (ТХА_2_) Prostaglandins H_2_ and G_2_	Nitric oxide (NO) Prostaglandin E_2_ (PGE_2_) Endothelium-derived hyperpolarizing factor (EDHF) Bradykinin C-type natriuretic peptide Adrenomedullin (ADM) Endothelin-3
*Hemostasis and antithrombosis regulators*	
Prothrombogenic factors	Antithrombogenic factors
Platelet-derived growth factor (PDGF) Plasminogen activator inhibitor-1 (PAI-1) Von Willebrand factor (coagulation factor VIII) Angiotensin IV Endothelin-1	NO Tissue plasminogen activator (tPA) Prostacyclin (PGI2)
*Leukocyte adhesion regulators*	
Adhesion stimulants (E-selectin, P-selectin, intercellular adhesion molecule-1 (ICAM-1)), vascular cell adhesion molecule-1 (VCAM-1)	
Vascular growth regulators	
Stimulants	Myocyte migration and proliferation inhibitors
*Regulators of inflammation, vascular permeability, and apoptosis of vascular wall components*	
Stimulants	Inhibitors
Tumor necrosis factor *α* (TNF-*α*) Superoxide radicals (О_2_^–^, OONO^–^) Protein kinase C (PKC)	NO

## Data Availability

Data can be available upon request.
